# Oral Tongue Malignancies in Autoimmune Polyendocrine Syndrome Type 1

**DOI:** 10.3389/fendo.2018.00463

**Published:** 2018-08-17

**Authors:** Øyvind Bruserud, Daniela-Elena Costea, Saila Laakso, Ben-Zion Garty, Eirik Mathisen, Antti Mäkitie, Outi Mäkitie, Eystein S. Husebye

**Affiliations:** ^1^Department of Clinical Science, University of Bergen, Bergen, Norway; ^2^K.G. Jebsen Centre for Autoimmune Disorders, University of Bergen, Bergen, Norway; ^3^Gade Laboratory for Pathology, Haukeland University Hospital, Bergen, Norway; ^4^Department of Clinical Medicine, University of Bergen, Bergen, Norway; ^5^Centre for Cancer Biomarkers, University of Bergen, Bergen, Norway; ^6^Children's Hospital, University of Helsinki and Helsinki University Hospital, Helsinki, Finland; ^7^Folkhälsan Institute of Genetics, Helsinki, Finland; ^8^Allergy and Immunology Clinic, Schneider Children's Medical Center of Israel, Tel Aviv, Israel; ^9^Sackler Faculty of Medicine, Tel Aviv University, Tel Aviv, Israel; ^10^Department of Otolaryngology-Head and Neck Surgery, Østfold Hospital, Sarpsborg, Norway; ^11^Department of Otorhinolaryngology, Head and Neck Surgery, University of Helsinki and Helsinki University Hospital, Helsinki, Finland; ^12^Department of Medicine, Haukeland University Hospital, Bergen, Norway

**Keywords:** Autoimmune polyendocrine syndrome type 1, oral malignancies, chronic mucocutaneous candidiasis, endocrinology, *Autoimmune Regulator* gene

## Abstract

Autoimmune polyendocrinopathy-candidiasis-ectodermal dystrophy (APECED) or Autoimmune polyendocrine syndrome type-1 (APS-1) (APECED, OMIM 240300) is a rare, childhood onset, monogenic disease caused by mutations in the *Autoimmune Regulator* (*AIRE*) gene. The overall mortality is increased compared to the general population and a major cause of death includes malignant diseases, especially oral and esophageal cancers. We here present a case series of four APS-1 patients with oral tongue cancers, an entity not described in detail previously. Scrutiny of history and clinical phenotypes indicate that chronic mucocutaneous candidiasis and smoking are significant risk factors. Preventive measures and early diagnosis are important to successfully manage this potentially fatal disease.

## Introduction

Autoimmune polyendocrinopathy-candidiasis-ectodermal dystrophy (APECED) or Autoimmune polyendocrine syndrome type-1 (APS-1) (APECED, OMIM 240300) is a rare, childhood onset, monogenic disease caused by mutations in the *Autoimmune Regulator* (*AIRE*) gene. It is clinically defined by the presence of two of the three main components: hypoparathyroidism (HP), primary adrenocortical insufficiency (PAI), and chronic mucocutaneous candidiasis (CMC) (1, 2), but several less known organ-specific manifestations are also part of the syndrome making the clinical phenotype highly variable (2–4). The overall mortality is increased (5, 6) due to different complications such as acute adrenal crisis (3, 5), severe pneumonitis with respiratory failure (7–9), fulminant autoimmune hepatitis (4), and interstitial nephritis causing renal failure (10, 11).

Malignancies are not uncommon in APS-1, and squamous cell carcinoma (SCC) of the oral or esophageal mucosa is the most common entity (3, 5). In a case series by Rautemaa et al., most patients were in their thirties and had metastatic disease at diagnosis (12). In one of the cases the carcinoma affected the tongue, and CMC and smoking were associated with the malignancies (12).

Oral malignancies typically develop in middle-aged or older individuals, often in the fifth or sixth decade of life, and there is a well-established association between both smoking and heavy drinking, and SCC (13–15). Other disorders affecting the oral and gastrointestinal mucosa, such as infections and atrophic gastritis, may also contribute to the development of malignancies (16, 17). Notably, oral leukoplakia has the potential of malignant transformation (18). Oral tongue SCC is the most common type of oral malignancies and its diagnosis is based on clinical examination combined with proper imaging using computer tomography (CT) or magnetic resonance imaging (MRI) together with histology of a tissue biopsy. The primary treatment approach for oral malignancies is wide surgical resection with clean margins, as marginal infiltration is associated with risk of recurrence and impaired survival (19, 20). Postoperative treatment typically consists of radiotherapy or chemo radiotherapy depending on the disease stage and surgical outcome.

Recently, we have come across several APS-1 patients diagnosed with oral tongue cancers, which seems to be a distinct entity associated with APS-1. Here we highlight their clinical presentation, diagnosis, treatment, and follow up. We also briefly discuss the biological aspects of oral malignancies in the context of APS-1.

## Background

Basic APS-1 characteristics of the patients including *AIRE-*mutations are summarized in Table 1. In the following text, we briefly describe each patient focusing on the onset, diagnosis, and treatment of their tongue SCC.

**Table 1:** Characterization of the APS-1 patients.

**Pat. no**.	**Sex**	**DoB**	**Age of onset**	**Classic triad**	**Other manifestations**	***AIRE* mutations**	**Autoantibodies**
1	F	1967	1	Hypoparathyroidism (1), Chronic mucocutaneous candidiasis (3), Primary adrenal insufficiency (16)	Enamel hypoplasia (6), hypogonadism (13), Vitiligo (13), alopecia (27), vitamin B12 deficiency (28), malabsorption (30), diabetes mellitus type 1 (31), asplenism (39), autoimmune thyroiditis (47)	R257X/R257X	SCC, NALP5, INF-ω
2	F	1965	2	Hypoparathyroidism (2), Primary adrenal insufficiency (5), Chronic mucocutaneous candidiasis (10)	Enamel hypoplasia (5), alopecia (10), hypogonadism (15), tubulointerstitial nephritis (19), autoimmune thyroiditis (32)	R257X/R257X	17OH, SCC, NALP5, IL22, INF-ω
3	M	1996	11	Primary adrenal insufficiency (11), Chronic mucocutaneous candidiasis	Hepatitis(0), malabsorption(0), asplenism	c.967_979del13/c.967_979del13	21OH, 17OH, AADC, IL22, SCC, TPH1, INF-ω
4	M	1970	3	Hypoparathyroidism (5), Primary adrenal insufficiency, Chronic mucocutaneous candidiasis	Alopecia(3), hepatitis, vitiligo, asplenism	A374G/A374G	21OH, TPO

*The age at diagnosis for each disease component is written in parentheses. The age of onset denotes the age at which the first APS-1 main component appeared. Abbreviations: 21OH, 21-hydroxylase; 17OH, 17-α-hydroxylase; AADC, aromatic L-amino acid decarboxylase; DoB, date of birth; IL22, interleukin-22; INF-ω, interferon-omega; NALP5, NACHT leucine-rich-repeat protein 5; Pat no, patient number; SCC, side-chain-cleavage enzyme; TPH1, tryptophan hydroxylase 1; TPO, thyroid peroxidase*.

### Patient #1

This Finnish female patient (born 1967) was diagnosed with HP at the age of 18 months. She presented with CMC in the mouth and esophagus from the age of three years; regular antifungal medication had not been used. The APS-1 diagnosis was established in early childhood by *AIRE* sequencing. She has smoked regularly from the age of 14 years (currently 1–4 cigarettes a day), but only consumed 4–5 units of alcohol per year.

At the age of 37 years endoscopic esophagus dilation was performed because of stenosis. At the age of 45 years she presented with a 1 cm ulceration on the right side of the tongue. Histology revealed locally invasive SCC of World Health Organization (WHO) Grade 1 without positive neck nodes (T1N0M0, Stage I). A radical resection was performed. No postoperative radiotherapy was given. She is now disease free after an uneventful five-years follow up.

### Patient #2

This Finnish female patient (born 1965) was diagnosed with HP at the age of two years and has had oral CMC since the age of 10 years. The APS-1 diagnosis was made based on clinical manifestations and confirmed by *AIRE* sequencing. Renal transplantation was performed at the age of 24 years because a tubulointerstitial nephritis causing end-stage renal failure. She presented with particularly severe CMC infections from the age of 40 years. The yeast was fluconazole and itraconazole resistant, but amphotericin B sensitive, and she received local treatment with this medication. She has never been a regular smoker and reported current alcohol use of about four units per week.

**Figure 1:**
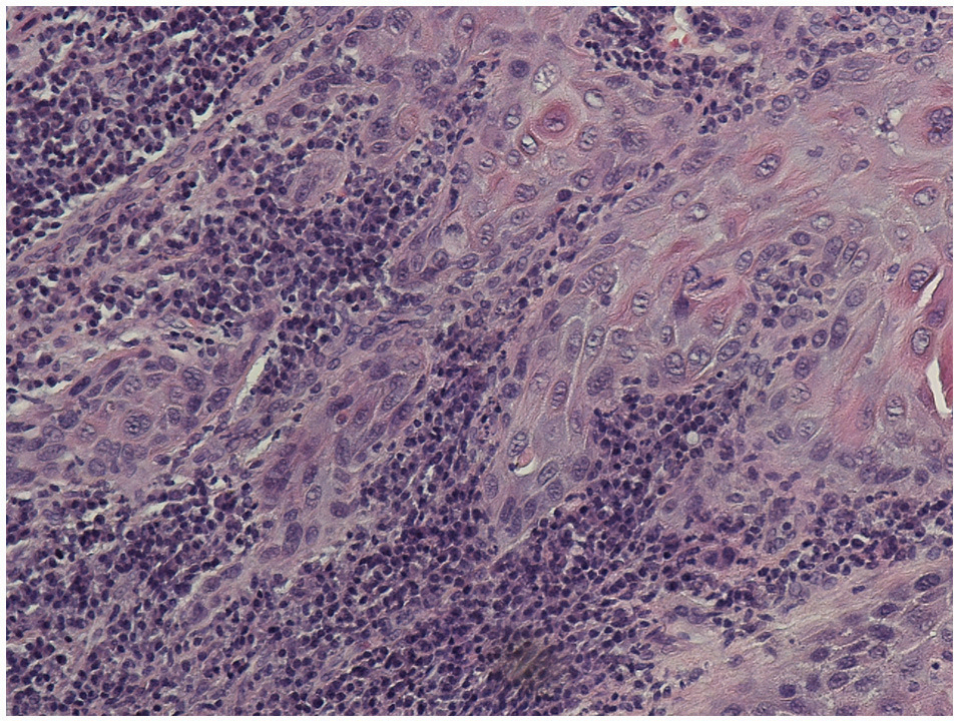
Invasive squamous cell carcinoma in the mobile tongue of patient #2 with APS-1 (x 200 magnification).Histological picture showed a well differentiated SCC with a rich lymphocytic inflammatory infiltrate at the tumor front.

At the age of 30 years she was diagnosed with carcinoma *in situ* of the right side of the tongue and a radical surgical resection was performed. However, a local recurrence of SCC (T1N0M0, Stage I) occurred one year after the initial treatment and a hemiglossectomy with a radial forearm free-flap reconstruction was performed (Figure 1). No postoperative radiotherapy was given. During follow up, several biopsies were taken revealing dysplastic changes including signs of SCC *in situ*. Seven years after the first diagnosis, microinvasive carcinoma was diagnosed in the right mandibular gingiva in the region of molar 46–47. The lesion was treated with photodynamic therapy (PDT) with 5 mm clinical margins. PDT was delivered as an alternative postoperative treatment option due to the history of recurrent multifocal SCC, assuming that repeated surgical resections and conventional radiotherapy would have caused a higher risk for further impaired oral function. During a 16-year follow up, two granulomatotic gingival lesions have been resected 13 years after the primary diagnosis; histology showed mild dysplasia, gingivitis and fungal infection.

### Patient #3

This Norwegian male patient (born 1996) presented with autoimmune hepatitis and severe malabsorption during the first year of life. He also had recurrent CMC infections in early childhood. PAI was diagnosed at 11 years of age. The APS-1 diagnosis was confirmed by *AIRE* sequencing. His gastrointestinal manifestations have been treated with mycophenolate mofetil and tacrolimus with a good response. The patient has also been diagnosed with asplenism. He neither smokes nor uses alcohol.

**Figure 2:**
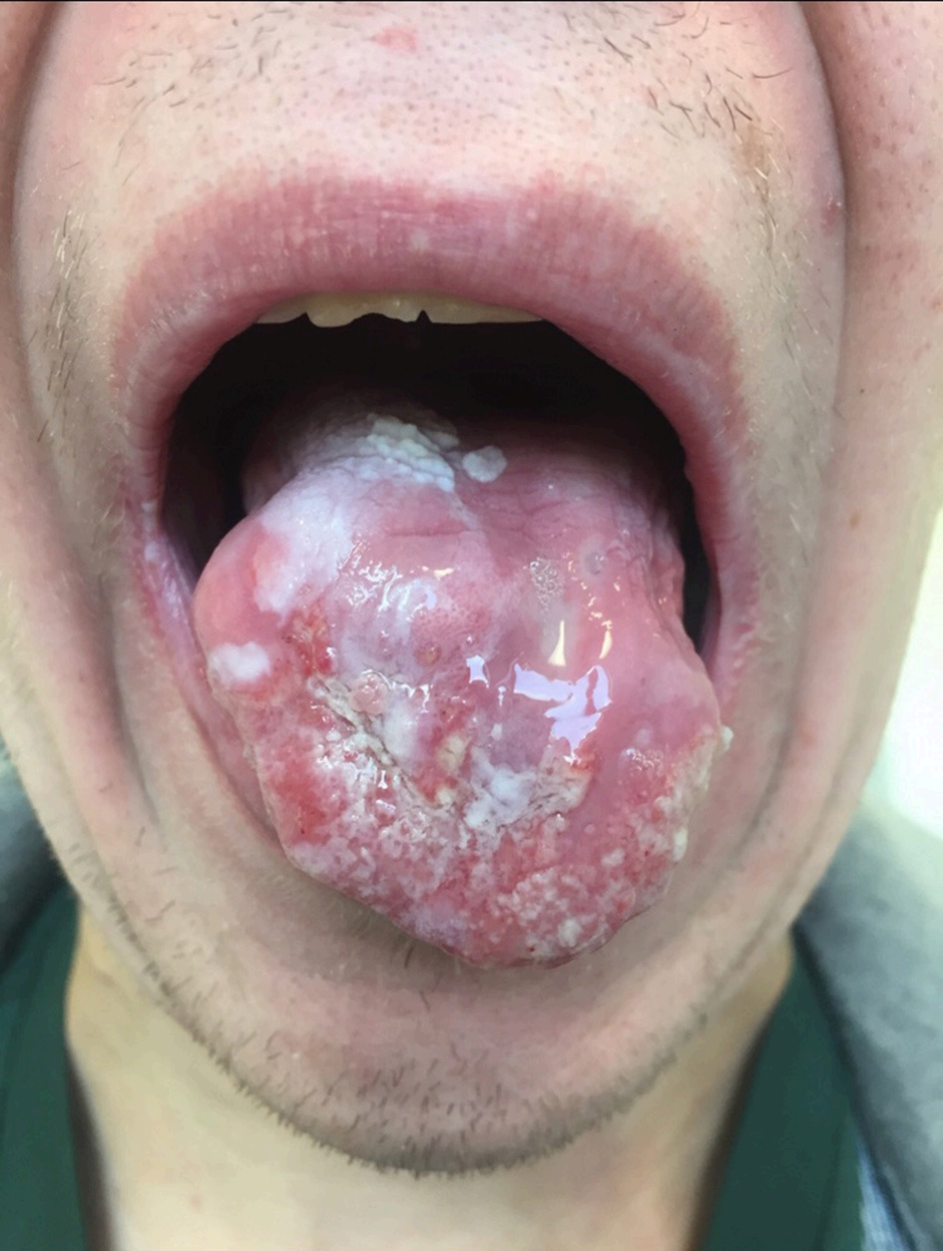
A picture of the tongue of patient #3 at time of diagnosis. The patient presented with severe CMC, glossitis, and severe pain in the tongue. Extensive, non-homogenous changes in the form of speckled leucoplakia were observed covering the whole dorsal side of the tongue which was sensitive and indurated at palpation and functionally compromised with limited movements.

**Figure 3:**
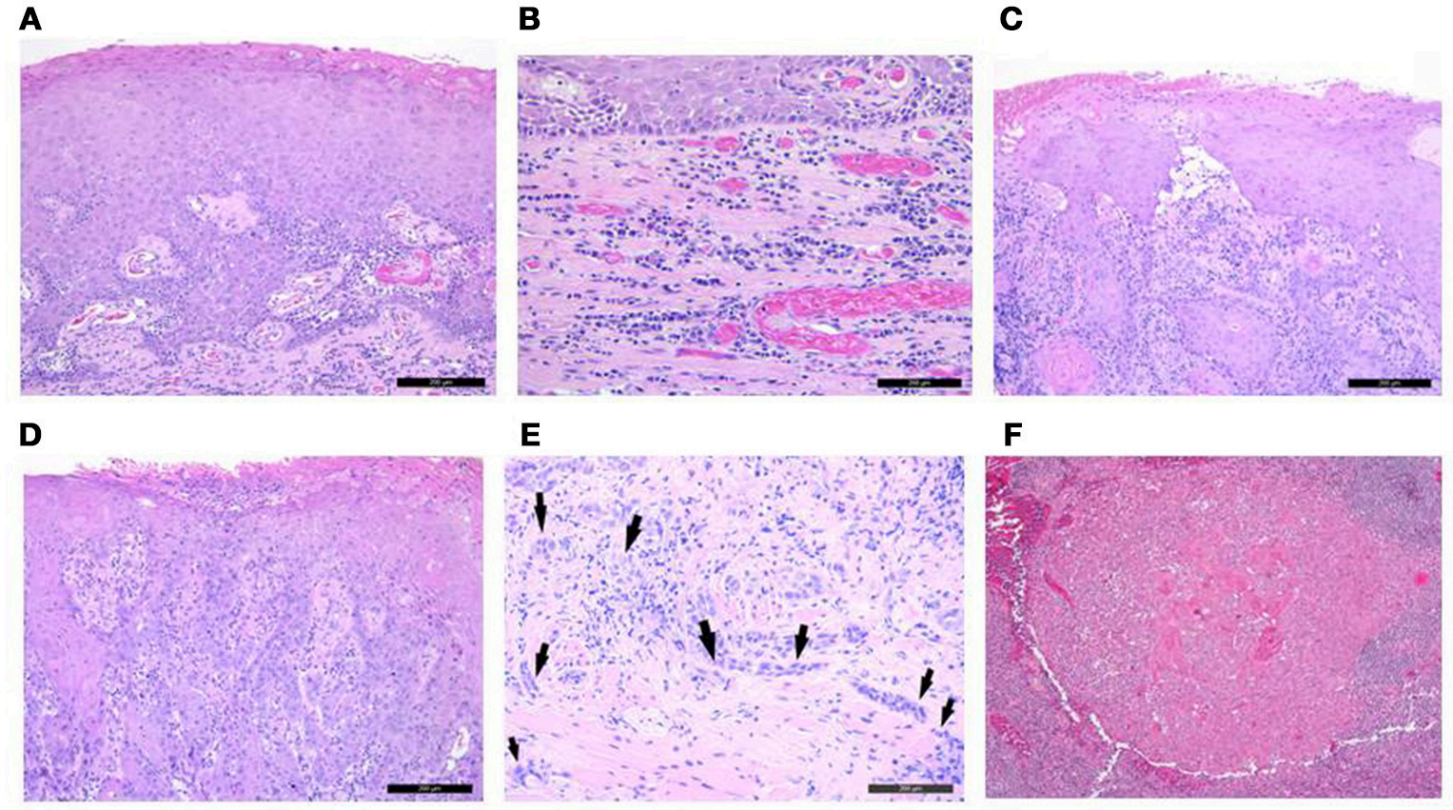
Histological images of several biopsies taken from the tongue of patient #3. **(A)** epithelial hyperplasia with hyperkeratosis (x 100 magnification); **(B)** stromal inflammation dominated of plasma cells (x 200 magnification); **(C)** well differentiated SCC (x 100 magnification); **(D)** poorly differentiated SCC (x 100 magnification); **(E)** Non-cohesive cancer foci, tumor cords, and single cells (black arrows) observed at the invasive front indicate a highly aggressive SCC lesion (x 200 magnification). Note the lymphocytic inflammatory infiltrate toward the more central area of the tumor, but its lack at the very edge of the invasive tumor front; **(F)** Histological analysis of the lymph nodes removed at the time of hemiglossectomy revealed squamous cell carcinoma metastasis spread to one lymph node (x 100 magnification).

At the age of 21 years he developed severe glossitis and pain in the tongue (Figure 2). A constantly elevated lymphocyte count in peripheral blood was also present. Initial biopsy revealed stromal inflammation and hyperkeratosis without signs of malignancy. However, the pain continued and, 2 months later, new biopsies showed areas with epithelial hyperplasia, hyperkeratosis (Figure 3A), and stromal inflammation dominated of plasma cells (Figure 3B), and invasive SCC with a various histologic appearance from well (Figure 3C) to poorly differentiated lesions (Figure 3D) at five different locations. The invasive tumor front showed non-cohesive cancer foci, tumor cords, and single cells, indicating an aggressively invasive lesion (Figure 3E). Hemiglossectomy and a reconstruction using a radial forearm free flap were performed. Moreover, investigation of the surgical specimen revealed metastasis into one lymph node (Figure 3F). The tumor was classified as T3N1M0, Stage III. He received postoperative cisplatin-based chemotherapy and radiotherapy because of an incomplete surgical resection and has no signs of residual disease after 7 months follow up.

### Patient #4

This male patient with APS-1 (born 1970) was the son of Persian Jews who were first cousins. He presented, at the age of three years, with alopecia areata, which advanced during the next 4 years to alopecia totalis. HP was diagnosed at the age of five. During the following years additional diseases developed including PAI, vitiligo, bilateral cataract, keratitis, pernicious anemia, hepatitis, and asplenism. He had CMC since childhood and had numerous episodes of oral and oesophageal candidiasis which was treated with nystatin, ketoconazole and fluconazole. There was no history of smoking or alcohol consumption.

At the age of 38 years, a 2 cm mass was observed on the right side of the tongue. Biopsy revealed SCC. Partial glossectomy with selective neck dissection was performed and postoperative radiotherapy to the primary site and to the neck was delivered. Unfortunately, two years later, the tongue tumor recurred. Chemotherapy was initiated, but without effect on tumor growth and the patient died a few months later due to *Staphylococcal aureus* septicaemia.

## Discussion

We here report four APS-1 patients with oral tongue SCC at a relatively young age treated with radical surgical resection alone or in combination with chemo-radiotherapy, or PDT in one case. All patients had severe CMC since childhood; some in combination with other well-known risk factors for oral malignancies such as smoking and alcohol use. Apart from early onset, the clinical presentation and histology of the tumors was similar to other patients with oral malignancies. Based on our findings, a prevalence of oral tongue malignancies in the entire APS-1 cohort can be estimated to about 1–2 percent. However, our case series highlights the importance of aggressive CMC treatment and regular follow up examination of the oral mucosa in APS-1 patients.

All four patients presented in childhood with CMC as part of their initial APS-1 manifestations, probably causing a longstanding chronic inflammation in the oral cavity. CMC is a common and early main manifestation of APS-1 (2, 21). It usually affects the oral mucosa as angular cheilitis or the whole mouth causing hypertrophic and/or atrophic lesions (4, 5, 21). In APS-1, the chronic inflammation of CMC changes the microenvironment of the oral cavity causing gingivitis and glossitis (3) and it is proposed that patients are almost persistently colonized with *Candida albicans* despite the relief of symptoms found in response to treatment (22). Oral mycostatin or oral amphotericin B is recommended to manage oral CMC in APS-1 to avoid the problem of drug resistance and the inhibition of steroidogenesis associated with continuous use of azole preparations (2). Studies in mice have suggested that autoreactive CD4+ T cells and chronic fungal infections cause inflammation and tissue injury, which further drive carcinogenesis (23). Although a synergistic effect of other risk factors such as smoking and alcohol use is possible, these factors were not prominent in two patients (#3; #4) and only one patient (#1) reported to smoke on a daily basis.

The third patient had severe CMC and glossitis. Initial biopsies did not reveal malignancy but stromal inflammation and hyperkeratosis. However, the clinical examination and visual findings gave a strong suspicion of malignancy and repeated biopsies showed SCC. This highlights the crucial role of the clinical examination including a visual inspection of the oral cavity which should be included in the regular surveillance of patients with APS-1. Using endoscopes with a “narrow-band imaging” modality to screen oral and pharyngeal mucosa should be considered in postoperative follow up and in high risk patients, as this tool seems to increase the detection rate of dysplastic and carcinoma lesions (24). This case also points out the importance of selecting the area for biopsy and that several biopsies might be necessary in case of extensive non-homogenous mucosal changes, as for this patient where biopsies from different areas showed a histological appearance varying from no dysplasia to poorly differentiated carcinoma (Figure 3). No general histologic feature predictive for carcinogenesis could be recognized. Nevertheless, the clinical suspicion for malignancies should be high to secure proper and timely diagnosis.

All our patients described here had typical APS-1 manifestations and disease-causing *AIRE* mutations. Recent studies have investigated the potential role of *AIRE* in cancer and malignancies. For example, the expression of AIRE protein has been verified in human breast cancer cells and seems to be a strong prognostic factor for relapse-free survival (25). Moreover, in human and mice keratinocytes, *AIRE* expression is inducible in a keratin 17-dependent manner which is required for timely onset of Gli2-induced skin tumorigenesis in mice (26). However, genome-wide gene expression profiling of tongue SCCs using RNA-sequencing has not revealed *AIRE* expression (27, 28). Another recent elegant study in mice showed that *Aire*-deficiency decreased the thymic expression of phosphoribosyl-anthranilate isomerase (TRP-1), which is a self-antigen in melanocytes and a cancer antigen in melanomas (29). This leads to defective negative selection of TRP-1-specific T cells and elevated T-cell immune responses that were associated with suppression of melanoma outgrowth (29). In addition, transplantation of *Aire*-deficient thymic stroma was sufficient to confer more effective immune rejection of melanoma in otherwise *Aire* wild-type hosts (29). Taken together, *AIRE* probably has functions beyond thymic negative selection of T cells and may play a role in the development of malignancies. This also underpins the value of APS-1 as a powerful model disease for studying immunological mechanisms in both autoimmunity and malignancies.

## Concluding remarks

Oral manifestations in APS-1 should be properly investigated to reveal initial signs of oral malignancies including tongue carcinomas. This malignancy seems to be a hitherto undescribed distinct entity associated with APS-1. CMC should be aggressively treated, and the risk factors reduced, to avoid development of oral malignancies in these patients. A regular investigation of the oral cavity is recommended as part of the annual follow up.

## Ethics statement

Written informed consent has been obtained from all patients alive and from the next of kin of the deceased patient for publication of the case report and accompanying images.

## Author contributions

All authors contributed in the clinical characterization of the patients and in writing and critically reviewing the manuscript. D-EC described the histologic pictures.

### Conflict of interest statement

The authors declare that the research was conducted in the absence of any commercial or financial relationships that could be construed as a potential conflict of interest.
